# Supplementary material for: Phylogenetic congruence of lichenised fungi and algae is affected by spatial scale and taxonomic diversity
Source: PeerJ. 2014 Sep 11;2:e573. doi: 10.7717/peerj.573 (PMC4168761; doi:10.7717/peerj.573)
Supplement: Table S1 — Raw genetic and geographic distance matrices for each dataset. [file peerj-02-573-s001.docx]

**Supplemental Table**: Results from the GenBank BLAST search of specimens in the Flock Hill community dataset. Despite there being no 100% matches to provide certain identification, ‘species’ are specimens that had a greater than or equal to 99% sequence match to a GenBank sequence that had a species name. ‘Genus level’ specimens are those that had a greater than 97% match to a named GenBank sequence. ‘Family level’ specimens in the same OTU are those that did not have greater than 97% match, but are ≥97% identical to each other.

| Identification | OTU | Identity (% match) | Number of specimens |
| --- | --- | --- | --- |
| Species | *Ramalina glaucescens* | 99 | 4 |
|  | *Austroparmelina norpruinata* | 99 | 1 |
|  | *Parmelia subtestacea* | 99 | 2 |
|  | *Myelochroa aurulenta* | 99 | 1 |
| Genus level | *Hypogymnia sp.* 1 | ≥97 | 13 |
|  | *Usnea* sp.1 | ≥97 | 8 |
|  | *Usnea* sp.2 | ≥97 | 1 |
|  | *Usnea* sp.3 | ≥97 | 4 |
|  | *Hypotrachyna* sp. 1 | ≥97 | 1 |
|  | *Lecanora* sp. 1 | ≥97 | 5 |
|  | *Lecanora* sp. 2 | ≥97 | 1 |
|  | *Melanelixia* sp. 1 | ≥97 | 7 |
| Family | Parmeliaceae OTU1 |  | 4 |
|  | Parmeliaceae OTU2 |  | 1 |
|  | Parmeliaceae OTU3 |  | 1 |
|  | Ramalinaceae OTU1 |  | 1 |
|  | Rhytismataceae OTU1 |  | 1 |
|  | Sarrameanaceae OTU1 |  | 1 |
|  | Teloschistaceae OTU1 |  | 1 |
|  |  |  | Total = 58 |
